# Development of a Predictive Model for Identifying High-Risk Older Adults for Geriatric Emergency Department Screening

**DOI:** 10.1016/j.acepjo.2026.100414

**Published:** 2026-05-22

**Authors:** Karen A. Hauser, Jeremy Swartzberg, David Schlessinger, Nasrin Samady, Karin Dove, Michelle Donnelly, Cristina D. Perkins, Dana Sax, Marlena Tang, Vincent X. Liu

**Affiliations:** 1 Department of Hospital Medicine, Kaiser Permanente San Francisco Medical Center, San Francisco, California, USA; 2 Department of Emergency Medicine, Kaiser Permanente Oakland Medical Center, Oakland, California, USA; 3 Division of Research, Kaiser Permanente Northern California, Pleasanton, California, USA; 4 Kaiser Permanente, Oakland, California, USA; 5 Kaiser Permanente, Pleasanton, California, USA; 6 Department of Emergency Medicine, Kaiser Permanente San Francisco, San Franicsco, California, USA

**Keywords:** geriatric emergency department, geriatric emergency department screening, high-risk geriatric patients, screening model

## Abstract

**Objective:**

Geriatric emergency department (GED) programs face challenges in risk-stratifying older adults. Existing tools, like the identification of seniors at risk (ISAR) score model, have modest predictive accuracy and are difficult to implement sustainably. We undertook a quality improvement initiative to develop, evaluate, and validate a screening score (SS) within a large, integrated health care system. This score, which can be deployed and automated at emergency department (ED) triage, identifies older adults at risk of subsequent ED or hospital use and short-term mortality.

**Methods:**

The GED-SS score model was developed from a multicenter cohort of ED patients aged ≥70 years with an Emergency Severity Index > 1 from January 1, 2018, until December 31, 2019. The composite outcome was ≥3 days of acute care (ED, observation, or inpatient) or death within 90 days. The GED-SS score model was prospectively validated in 1313 ED patients and then compared with the nurse-performed ISAR score screenings.

**Results:**

The GED-SS score model showed better discrimination with an area under the curve (AUC) value of 0.73 vs 0.66 for the ISAR score model identification. At a 43% sensitivity threshold (based on an ISAR score model cutoff value of ≥ 3), the GED-SS score model had higher specificity (86% vs 78%) and positive predictive value (59% vs 47%), while also flagging fewer patients (23.3% vs 29.1%).

**Conclusion:**

We developed and validated a GED-SS score model to identify older adult ED patients at increased risk of short-term mortality and acute care utilization. The model, which uses structured data facilitating automatic calculation, was prospectively validated and performed comparably to or better than the ISAR score model.

**The Bottom Line**

Kaiser Permanente Northern California developed an automated screening score to identify emergency department (ED) patients aged ≥70 years who would benefit from specific geriatric ED care pathways. The score is automated, can be run on all elderly patients in the ED at triage, and works better than the identification of seniors at risk score to predict ED or hospital visits and mortality.

## 1 Introduction

### 1.1 Background

Older adults account for up to 1 in 4 emergency department (ED) visits and are at greater risk for adverse outcomes, including functional decline, ED return visits, hospitalization, and death.^1^ Geriatric EDs (GEDs) have emerged as a model of care to identify, prevent, and treat key geriatric syndromes to improve patient outcomes.^2^ GEDs began appearing in the United States in 2007, with standardized GED accreditation criteria adopted by the American College of Emergency Physicians in 2017, and more than 600 EDs now achieving accreditation.^3^, ^4^, ^5^, ^6^ GED programs are associated with a reduced likelihood of hospital admission and reutilization as well as improved care costs.^7^, ^8^, ^9^, ^10^, ^11^, ^12^, ^13^, ^14^

Current GED guidelines recommend that all older adults be screened for geriatric syndromes during their ED visit to identify patients who may benefit from additional resources and interventions.^15^ Several prognostic tools have been developed to identify older adults at high risk of adverse outcomes following an ED visit, including the identification of seniors at risk (ISAR) score model.^16^ The ISAR score screen consists of 6 yes/no self-report questions related to functional dependence, recent hospitalization, impaired memory and vision, and multiple medications with scores ranging from 0 to 6.^17^ This tool uses cutoff points of ≥ 2 to identify higher-risk patients who might benefit from tailored GED interventions, with a threshold of ≥ 3 exhibiting a higher specificity and lower sensitivity.^18^ The ISAR score model’s reliability and predictive validity for a range of adverse outcomes, including functional decline, mortality, hospitalization, and unplanned hospital or ED revisit, have been well-examined.^19^ However, the ISAR score model exhibits highly variable model discrimination, with area under the receiver operating characteristic curve (AUC) values ranging from 0.51 to 0.80 for ED revisit, hospitalization, or death.^20^ Despite its simplicity, the ISAR score model has been described as difficult to implement, with low screening compliance rates among frontline ED staff, which was replicated in our pilot testing.^16^

### 1.2 Importance

Not all older adult patients can undergo a comprehensive geriatric assessment, given resource and time constraints in the ED. Thus, GED programs face a challenge in accurately and sustainably risk-stratifying older adults to focus resources on those most in need of targeted geriatric services. Additional studies have explored automated screens using available electronic health record (EHR) data, though their performance compared with existing validated tools is unknown.^21^^,^^22^ There remains a need for validated and automated screening instruments to identify patients for GED care.

### 1.3 Goals of This Investigation

We undertook a quality improvement initiative across Kaiser Permanente Northern California (KPNC) to retrospectively develop and evaluate a screening score that could be implemented at ED triage without additional data entry and identify high-risk older adult patients who might benefit from GED services. We designed this effort as a quality improvement initiative because our GED program needed complementary screening tools that could identify patients who would potentially benefit from GED intervention, would reduce manual inputs, and could be tied to workflow enhancements. We then assessed the model’s performance score prospectively and compared its performance to the ISAR score, which was used in pilot testing at our facility.

## 2 Methods

KPNC is an integrated health care delivery system with 21 medical centers, serving >4.5 million members with demographics comparable to the broader patient population in the region.^23^, ^24^, ^25^ Analyses were conducted in this study as part of a regional GED quality improvement program with an initial pilot site at KP San Francisco Medical Center, with plans to implement GED program elements at all remaining medical centers. This quality improvement analytic approach, reviewed by the KPNC Research Determination Committee, was deemed exempt from KPNC Institutional Review Board review (RDO KPNC 22 – 044, November 9, 2022).

### 2.1 GED-SS Score Model Development Cohort

The GED score development cohort included all patients presenting to the ED at 21 KPNC hospitals aged ≥70 years, who had active KP membership at ED arrival, and had an Emergency Severity Index (ESI)^26^ score > 1 between January 1, 2018, and December 31, 2019. We included only KP health plan members to reduce bias from missing predictor and outcome data. We excluded patients with an ESI score of 1 because they require immediate life-saving interventions and would be unlikely to receive routine GED interventions.

### 2.2 Outcome

We defined the outcome as 3 or more days of care in a hospital setting (ED, observation, or inpatient care) or death in the 90 days following an ED encounter. This composite outcome was chosen because it (1) represents consequences of untreated geriatric syndromes that GED interventions aim to prevent, (2) has been validated in prior ISAR score model studies to allow direct comparison, and (3) reflects a pragmatic objective of our GED program. Hospital days among patients admitted directly from their initial ED encounter could count toward this total. We assessed outcome data using longitudinal records within KPNC’s EHR, where patients are assigned a single medical record number to link records across all medical centers and databases.

### 2.3 Variables

Our goal was to use the GED-SS score model variables immediately available at ED arrival to enable automated, rapid identification of high-risk GED patients at ED triage. We chose not to include predictor data available during the triage process or ED care (eg, laboratory results, vital signs, and ED diagnoses) because initial screening would result in downstream workflows within the GED program, and patients not initially screened by the GED-SS score could still be eligible to receive GED care. Predictors were selected based on existing literature, clinician consensus, and availability within structured EHR fields. The model included the following predictors: age; a scalar comorbidity metric (Comorbidity Point Score [COPS2] version 2,)^27^^,^^28^; an abbreviated Laboratory Acute Physiology Score (abLAPS) based on laboratory results in the prior 30 days^23^ categorized into 4 levels (high, medium, low, and missing); number of ED “treat and release” days in the prior 90 days; number of inpatient days in the prior 90 days; number of medications filled at KP pharmacies in the prior 90 days; most recent Schmid Falls Risk score^29^; most recent physical function assessment score (physical function 5 prior level of function scale)^27^^,^^30^; history of specific EHR diagnosis and condition groupers for dementia, delirium, and prior substance abuse; social determinant of health flags related to financial insecurity, family support, and social support based on International Classification of Disease z-codes (Supplementary Appendix 1); use of the online kp.org patient portal; existing assignment of a KPNC primary care physician; high-risk medication use within the past 90 days based on a modified Beers criteria^31^ (Supplementary Appendix 2); and neurology department outpatient visit in the previous 90 days.

### 2.4 GED-SS Score Model Development

We randomly divided the data set into 67% training and 33% test (Supplementary Appendix 3). We evaluated several approaches to GED-SS score model development in the training set, including linear models, gradient boosted machines (GBMs), and automated machine learning based on software packages AutoML and Driverless AI from H2O. Logistic regression, GBM, and AutoML model development were performed using H2O version 3.34.0.7 via Python (v2.7.5). Driverless AI models were developed using the default settings of version 1.10.1.3. We report performance results of models trained in the training set and examined in the test set. All reported model metrics are based on the test set. We assessed model performance based on the AUC values, sensitivity, specificity, positive predictive values (PPVs), and percent of patients flagged at specific risk threshold values. Because our goal was to facilitate real-time EHR implementation, we selected a simpler model if the performance between algorithms was similar. Subsequent physician decisions about ED disposition (ie, whether a patient was admitted to the hospital) were unknown, as the GED-SS score model was designed for deployment at ED triage. In sensitivity analyses, we assessed model performance among patients who were admitted separately from those discharged. We also examined performance by subgroups including race/ethnicity, age group, and sex. Additional sensitivity analyses evaluated the model performance in predicting the individual outcome components of 90-day mortality and 3 or more days of care in a hospital setting.

### 2.5 Prospective GED-SS Score Model Validation and Comparison With ISAR Score Model

After retrospectively assessing model performance, we prospectively validated the GED-SS score model at our pilot medical center using EHR data and compared its performance to the ISAR score model completed by triage nurses during GED operational testing. This independent, prospective validation cohort included 1313 patients admitted to the ED between November 18, 2021, and January 27, 2022, and was based on the model performance metrics described above. To compare the GED-SS score model performance to the ISAR score model, we selected a GED-SS score threshold exhibiting the same sensitivity for our outcome as an ISAR score of ≥ 3. This ISAR score cutoff value was used, as a cutoff value of ≥ 2 resulted in too high a volume of patients for our GED care model. The prospective analysis was completed using SAS 9.04. Data are reported as mean ± SD or number (%).

## 3 Results

The model derivation cohort included 395,992 patients aged ≥70 years with ED visits in 2018 or 2019, with a mean age of 80.6 ± 7.4 years (Table 1). Patients experienced a mean of 2.2 ED or hospital days and 0.6 ED treat-and-release visits in the 90 days prior to ED arrival. A similar proportion had a prior diagnosis of dementia (n = 84,820, 21.4%) and delirium (n = 81,678, 20.6%). A total of 127,404 (32.2%) experienced ≥3 days in the ED or hospital or died in the 90 days following their ED arrival (8.9% death and 28.7% utilization).

**Table 1:** Development and prospective cohort baseline characteristics and outcomes among patients.

Characteristic	Retrospective development cohort (N = 395,992)	Prospective evaluation cohort (N = 1313)
	Mean (± SD)	
Age, y	80.6 (7.4)	80.4 (7.1)
COPS2 score	45.1 (38.6)	42.7 (38.2)
No. of ED + hospital days in previous 90 d	2.2 (4.8)	1.9 (4.5)
No. of ED treat and release in the previous 90 d	0.55 (1.6)	0.30 (0.8)
No. of medication fills in the previous 90 d	8.5 (6.9)	7.7 (6.5)
	N (% of total)	
Sex		
Female	225,452 (56.9)	661 (50.3)
Male	170,537 (43.1)	652 (49.7)
Unknown	3 (0.0)	0 (0.0)
Race/ethnicity		
Asian	39,411 (10.0)	446 (34.0)
White	241,132 (60.9)	610 (46.5)
Hispanic	54,727 (13.8)	121 (9.2)
Black/African American	32,733 (8.3)	123 (9.4)
Other	27,989 (7.1)	13 (1.0)
AbLAPS, laboratory acuity score.		
High	75,395 (19.0)	228 (17.4)
Low	31,323 (7.9)	85 (6.5)
Medium	30,309 (7.7)	74 (5.6)
Missing	258,965 (65.4)	926 (70.5)
History of dementia		
No	311,172 (78.6)	990 (75.4)
Yes	84,820 (21.4)	323 (24.6)
History of delirium		
No	314,314 (79.4)	1158 (88.2)
Yes	81,678 (20.6)	155 (11.8)
High-risk Medications^a^		
No	335,228 (84.7)	1162 (88.5)
Yes	60,764 (15.3)	151 (11.5)
kp.org patient portal use		
Active	282,439 (71.3)	1025 (78.1)
Not active	113,553 (28.7)	288 (21.9)
Outpatient neurology clinic visit in the previous 90 d		
No	377,668 (95.4)	1260 (96.0)
Yes	18,324 (4.6)	53 (4.0)
PCP assigned		
No	6820 (1.7)	65 (5.0)
Yes	389,172 (98.3)	1248 (95.1)
PLOF		
Able to walk	125,190 (31.6)	274 (20.9)
No score	250,828 (63.3)	962 (73.3)
Unable to walk	19,974 (5.0)	77 (5.9)
Schmid falls risk^29^		
High fall risk	69,461 (17.5)	274 (20.9)
Low fall risk	168,661 (42.6)	962 (73.3)
No score	157,870 (39.9)	77 (5.9)
Prior substance abuse		
No	377,885 (95.4)	1,232 (93.8)
Yes	18,107 (4.6)	81 (6.2)
Social determinants of health: any flag^b^		
No	392,218 (99.0)	1308 (99.6)
Yes	3774 (1.0)	5 (0.4)
Social determinants of health: family flag^b^		
No	394,842 (99.7)	1312 (99.9)
Yes	1150 (0.3)	1 (0.1)
Social determinants of health: finances flag^b^		
No	394,713 (99.7)	1311 (99.8)
Yes	1279 (0.3)	2 (0.2)
Social determinants of health: social support flag^b^		
No	394,561 (99.6)	1,311 (99.8)
Yes	1,431 (0.4)	2 (0.2)
Outcome: 3 or more contact days or death in the next 90 days		
No	268,588 (67.8)	894 (68.1)
Yes	127,404 (32.2)	419 (31.9)

abLAPS: Laboratory Acute Physiology Score; COPS2, Comorbidity Point Score; ED, emergency department; KP, Kaiser Permanente; PCP, primary care physician; PLOF, prior level of function.

a High-risk medications were defined based on a subset of Beers Criteria medications, including antipsychotics and benzodiazepines (Supplementary Appendix 2), prescribed or active in the 90 days preceding the ED visit.

b Social determinants of health flags were based on International Statistical Classification of Diseases and Related Health Problems 10th Revision codes grouped into categories of family, finances, and social support (Supplementary Appendix 1).

### 3.1 Model Performance

**Figure 1:**
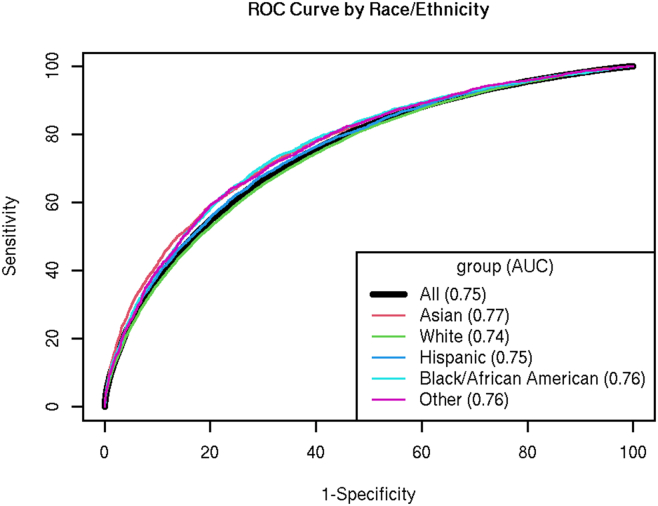
Receiver operating characteristic (ROC) curves using race/ethnicity for logistic regression model.

Standard logistic regression modeling without interaction terms exhibited an AUC value of 0.75 in the test set (Table 2). More advanced machine learning models exhibited similar or modestly improved performance, including GBM (0.75), AutoML (0.76), and Driverless AI (0.75). At a PPV threshold of 50%, which translates into a number needed to evaluate (NNE value − 1 divided by the PPV value) of 2:1, the models exhibited similar sensitivity (70%-71%) and percentages of flagged ED patients (44.7%-45.6%) (Table 2). The calibration curves showed good calibration for all the candidate models (Supplementary Appendix 4). Model performance across racial/ethnic, sex, and age subgroups was broadly similar (Fig 1 and Supplementary Appendix 5).

**Table 2:** Mode performance results.

Model type	AUC	NNE	Sensitivity	PPV	Flag %
Logistic regression	0.748	2.0	0.70	0.5	44.7%
GBM	0.751	2.0	0.71	0.5	45.3%
AutoML	0.755	2.0	0.71	0.5	45.6%
Driverless AI	0.752	2.0	0.71	0.5	45.5%

AUC, area under the curve; AutoML, Auotmated Machine Learning; Driverless AI, Driverless Artificial Intelligence; GBM, gradient boosted machine; NNE, number needed to evaluate; PPV, positive predictive value.

Because the logistic regression had similar results to the more complex machine learning models, we implemented the logistic regression model. We did not test for collinearity of predictors because clinical and operational partners determined all the candidate predictors to be important to include in the final model. Supplementary Appendix 6 shows a variety of model thresholds that were examined. To meet operational specifications, a threshold of 0.4 was chosen to identify those who might benefit from specialized GED care.

**Figure 2:**
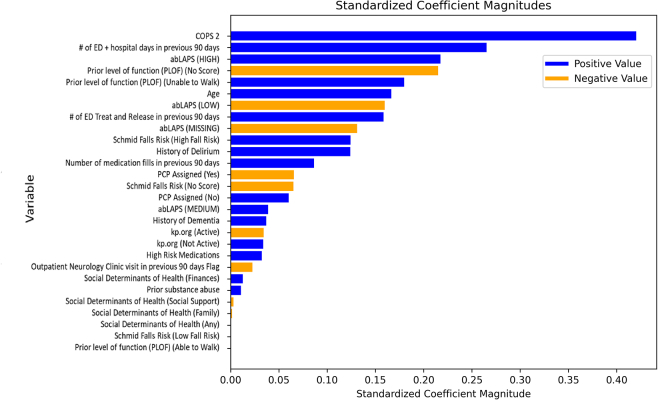
Logistic regression model coefficients. Beta coefficients computed when fit to standardized variables.

The logistic regression model score was strongly driven by the COPS2 score, number of prior ED visits and hospitalizations, and the abLAPS acuity score (Fig 2). For patients admitted to the hospital from the ED, the logistic regression model had an AUC value of 0.69 with a sensitivity of 68%, an NNE value of 1.4, and a flagged rate of 58.0% using the threshold described above. For patients who were not admitted, the logistic regression model had an AUC value of 0.77 with a sensitivity of 72%, an NNE value of 2.8, and a flagged rate of 39.0%. The AUC value was 0.76 for mortality alone and 0.73 for ≥3 days of acute care utilization. In the test set, 8.9% of encounters had a patient death within 90 days, and 28.6% had ≥3 days of acute care utilization (compared with 32.1% for the combined outcome).

### 3.2 Prospective Validation and Comparison With the ISAR Score Screening Tool

Patients in the independent, prospective cohort were of similar age to the training cohort but experienced fewer ED and hospital visits in the 90 days prior to ED admission than the model training cohort (1.9 vs 2.2 ED and hospital days, respectively; Table 1). Prospective validation of the GED-SS score screening tool exhibited an AUC value of 0.73, compared with 0.75 in the initial test set. The AUC value of the ISAR score screening tool in this cohort was 0.66. A high-risk threshold of ISAR score ≥ 3 resulted in a sensitivity of 43%. At a similar GED-SS score sensitivity threshold, the GED-SS score screening model exhibited higher specificity (86% vs 78%) and PPV value (59% vs 47%) while flagging fewer patients (23.3% vs 29.1%; Table 3). The ultimate operational GED-SS score threshold of 0.4 was slightly lower to optimize operational metrics like the percentage identified by the model as high risk.

**Table 3:** Model evaluation comparison: ISAR vs GED-SS sores, each evaluated at the threshold that results in a sensitivity of 43% (equivalent to ISAR score ≥ 3)

Variable	ISAR	GED-SS
AUC value	0.66	0.73
Sensitivity	0.43	0.43
PPV value	0.47	0.59
NPV value	0.74	0.76
Specificity	0.78	0.86
NNE value	2.11	1.69
% Flag	0.29	0.23

AUC, area under the curve; GED-SS, geriatric emergency department screening score; ISAR, identification of seniors at risk; NNE, number needed to evaluate; NPV, negative predictive value; PPV, positive predictive value.

## 4 Limitations

The GED-SS score screening tool may lack generalizability beyond the KPNC health system, as we excluded patients who were not KP health plan members due to the potential for incomplete data. However, the exclusion of non-KP members from model development did not preclude them from receiving GED interventions, as clinicians could manually identify patients who may benefit from GED care. An understanding of how the GED-SS score screening tool performs in patients with more limited EHR data (such as those lacking KP membership) is needed. Another important limitation is that we chose a utilization-based composite outcome for risk stratification, which may incompletely represent the comprehensive patient-centered outcomes valued by older adults treated in the ED. Our choice reflected what has been evaluated in prior studies, including those describing the ISAR scores and the anticipated sequelae of untreated geriatric syndromes, but is likely only 1 metric in a complex milieu of other potential outcomes. Further research and model validation should identify optimal patient-centered outcomes amenable to improvement through GED intervention and assess how the GED-SS score model performance can be enhanced. Finally, we had limited social determinants of health structured data, which would likely improve model performance and influence interventions.

## 5 Discussion

Using a large database of ED encounters of older adults within an integrated health system, we sought to develop and validate an automated screening score to identify older adults at high risk of acute care utilization and mortality who may benefit from GED care. Strengths of our model development included the large, multicenter patient cohort with robust longitudinal follow-up and subsequent prospective validation and comparison against the frequently used ISAR score screening tool. We examined the ISAR score screening tool because it is the most common screening tool used to identify older adults at risk of adverse outcomes in the ED.^17^^,^^18^^,^^32^ Many additional instruments have been described, including: revised ISAR (ISAR-R) score screening tool,^33^ Triage Risk Screening Tool,^27^ Variables Indicative of Placement Risk,^34^ the Silver Code,^35^ Mortality Risk Index,^36^ Program of Research to Integrate Services for the Maintenance of Autonomy—7 items (PRISMA-7) tool,^37^ Rowland,^38^ and Runciman Questionnaire.^39^ However, these screening instruments remain less studied and do not exhibit better simplicity or performance than the ISAR score screening tool.^40^^,^^41^

The GED-SS score demonstrated an AUC value of 0.75 in the retrospective test set and an AUC value of 0.73 in the prospective cohort, whereas the ISAR score ≥ 3 exhibited an AUC value of 0.66 in the prospective data collection.^42^ A meta-analysis of 32 ISAR score screening tool validation studies found the ISAR score had only modest discrimination for predicting 3-month ED revisits (AUC values, 0.57-0.66), hospitalizations (AUC values, 0.54-0.62), or death (AUC values, 0.53-0.62), consistent with our findings.^20^ An ISAR score threshold of ≥ 3 has been examined in prior studies, as this higher cutoff exhibits a better balance between sensitivity and specificity. ISAR score sensitivity at this threshold has been described as between 44% and 64% with a specificity of 49% to 80% for various adverse outcomes, consistent with the 43% sensitivity and 78% specificity of the ISAR score in our prospective validation cohort.^19^^,^^43^, ^44^, ^45^ Other model performance metrics (eg, specificity, sensitivity, and PPV values) were similar or better when comparing the GED-SS score with the ISAR score, although performance was lower among patients not admitted from the ED. Compared with the ISAR score screening tool, the GED-SS score screening tool demonstrated better performance and efficiency. It also allows for more precise adjustment of the flag threshold to meet the specific needs and capabilities of different GED programs, as it uses decimal probabilities instead of the ISAR score screening tool’s 6-point scale.

Although the GED-SS score exhibited better performance characteristics compared with the ISAR score, its model discrimination in patients discharged from the ED was only fair, suggesting future improvements can be made in identifying relevant patients. Because the GED-SS score screening tool was designed to be calculated at triage, patient disposition is not yet determined, complicating model development tailored to ultimate disposition. If the focus of GED programs were solely on patients expected to be discharged, model development could be weighted toward identifying the highest-risk patients among those also predicted to be discharged. However, GED programs still have a role in patients likely to be hospitalized to prevent downstream sequelae of ED care or even to prevent hospital admission entirely. In this initial development, we focused on a universal model calculated on all patients to serve as an initial screening tool.

Although predictive accuracy of screening instruments is important,^46^ implementation and feasibility are essential for effectiveness and sustainability.^47^ This is especially relevant in the fast-paced environment of emergency care. Recent studies have focused on electronic frailty indices or other automated EHR models, including those targeting specific geriatric syndromes or using machine learning algorithms, given their implementation benefits.^22^, ^48^, ^49^, ^50^, ^51^, ^52^, ^53^, ^54^ Barriers to successful implementation of traditional screening tools like the ISAR score include time required, insufficient resources, staff training, competing demands, and perceptions of the tools as superficial and poorly adapted to the local context.^19^^,^^27^^,^^32^^,^^55^^,^^56^ In prior studies, ISAR score completion rates by ED staff ranged from 17% to 54%, highlighting challenges with its utilization.^32^^,^^56^ By contrast, the GED-SS score utilizes existing structured data, allowing automatic calculation within the EHR during ED triage without the need for data entry to deploy the score or see the results. This approach addresses the sustainability concerns that have limited manual screening instruments while maintaining or improving predictive performance.

Given the complexity of clinical issues related to the care of vulnerable older adults, screening tools such as the ISAR or GED-SS score model are intended to augment but not replace clinical judgment.^18^^,^^57^ As a result, our GED program allows the clinical team to manually identify additional patients for GED care, and initial identification by either method is followed by further geriatric assessments based on the 5Ms (mind, mobility, medications, multicomplexity, and what matters most).^58^^,^^59^ The GED-SS score model was designed to integrate with GED clinical workflows and to be actionable at the point of triage, enabling real-time care pathway activation. Further study should evaluate whether GED interventions triggered by the GED-SS score screening tool reduce both geriatric syndrome burden and the downstream outcomes used in our model development.

In conclusion, we developed and validated a GED-SS score model to assist in identifying older adults treated in the ED who are at increased risk of subsequent ED and hospital utilization and short-term mortality. The model, which used structured EHR data with automated calculation at ED triage, exhibited comparable or improved performance to the ISAR score model. Future approaches should focus on integrating and improving screening tools within clinical pathways to improve the effectiveness, efficiency, and sustainability of GED programs.

## Author Contributions

KAH, JS, and VXL conceived the idea and drafted the manuscript. VXL and DS performed the analysis. RS and KD pulled the data. VXL coordinated data transfer and RDO submission. VXL and DS provided statistical consultation and helped to interpret the results. KAH, JS, DS, MT, and VXL provided clinical consultation. KAH, JS, DS, MT, and VXL contributed to the study design. VXL and DS oversaw the analysis and interpreted the results. All authors critically reviewed the manuscript.

## Funding and Support

This work was supported by a Dolby Family Foundation grant for geriatric emergency department development and The Permanente Medical Group. The funders had no access to the data and had no role in the design of the study; in the collection, analysis, or interpretation of data; in the writing of the manuscript; or in the decision to submit the manuscript for publication. David Schlessinger and Vincent X. Liu had full access to the data and take responsibility for the accuracy of the analysis.

## Conflict of Interest

All authors have read and approved the manuscript. All assume responsibility for its content. The authors report no conflicts of interest. The work is not under consideration elsewhere for publication.
