# Supplementary material for: Development of a Predictive Model for Identifying High-Risk Older Adults for Geriatric Emergency Department Screening
Source: J Am Coll Emerg Physicians Open. 2026 May 22;7(4):100414. doi: 10.1016/j.acepjo.2026.100414 (PMC13217493; doi:10.1016/j.acepjo.2026.100414)
Supplement: Supplementary Material [file mmc1.docx]

Development of a Predictive Model for Identifying High-Risk Older Adults for Geriatric Emergency Department Screening

Karen A Hauser, MD MPH; Jeremy Swartzberg, MD; David Schlessinger, MS; Nasrin Samady, MS; Karin Dove, MPH; Michelle Donnelly, BA; Cristina D. Perkins, JD, RN, MSN; Dana Sax, MD MPH; Marlena Tang, MD; Vincent X Liu, MD MSc

SUPPLEMENTARY MATERIAL

**Contents**

**Supplementary Appendix 1:** Social Determinant of Health ICD-10 Codes and Model Classification groupers

**Supplementary Appendix 2:** Definition of High-Risk Medications

**Supplementary Appendix 3:** Demographics of Training and Test Sample

**Supplementary Appendix 4:** Calibration plots for each model based on Test dataset

**Supplementary Appendix 5:** Results by Race/Ethnicity, Age Group, and Sex

**Supplementary Appendix 6:** Deciles of Thresholds for Logistic Regression Model (Test dataset)

**Supplementary Appendix 1:** Social Determinant of Health ICD-10 Codes and Model Classification groupers

| **GED Model Classification** | **Sub Classification** | **ICD10 Code** | **Description** |
| --- | --- | --- | --- |
| Finances | Finance | Z59.5 | Extreme poverty |
| Finances | Finance | Z59.6 | Low income |
| Finances | Finance | Z59.7 | Insufficient social insurance and welfare support |
| Finances | Finance | Z59.8 | Other problems related to housing and economic circumstances |
| Finances | Finance | Z59.9 | Problem related to housing and economic circumstances, unspecified |
| Finances | Housing | Z59.0 | Homelessness |
| Finances | Housing | Z59.1 | Inadequate housing |
| Finances | Housing | Z59.3 | Problems related to living in residential institution |
| Finances | Housing | Z59.9 | Problem related to housing and economic circumstances, unspecified |
| Finances | Housing | Z77.011 | Contact with and (suspected) exposure to lead |
| Finances | Housing | Z77.120 | Contact with and (suspected) exposure to mold (toxic) |
| Finances | Education | Z55.0 | Illiteracy and low-level literacy |
| Finances | Education | Z55.8 | Other problems related to education and literacy |
| Finances | Education | Z55.9 | Problem related to education and literacy, unspecified |
| Finances | Food | Z59.4 | Lack of adequate food |
| Finances | Employment | Z56.0 | Unemployment, unspecified |
| Finances | Employment | Z56.1 | Change of job |
| Finances | Employment | Z56.2 | Threat of job loss |
| Finances | Employment | Z56.3 | Stressful work schedule |
| Finances | Employment | Z56.4 | Discord with boss and workmates |
| Finances | Employment | Z56.5 | Uncongenial work |
| Finances | Employment | Z56.6 | Other physical and mental strain related to work |
| Finances | Employment | Z56.89 | Other problems related to employment |
| Finances | Employment | Z56.9 | Unspecified problems related to employment |
| Family | Family | Z63.4 | Disappearance and death of family member |
| Family | Family | Z63.6 | Dependent relatives needing care at home |
| Family | Family | Z63.79 | Other stressful life events affecting family and household |
| Social Support | Social Support | Z60.2 | Living alone |
| Social Support | Social Support | Z60.4 | Social exclusion and rejection |
| Social Support | Social Support | Z63.8 | Other specified problems related to primary support group |
| Social Support | Social Support | Z63.9 | Problem related to primary support group, unspecified |

**Supplementary Appendix 2:** Definition of High-Risk Medications

A patient was flagged for high-risk medications if they had any of the following medications prescribed in the outpatient or inpatient settings in the 90 days before the ED encounter. These medications were derived from a subset of the Beers Criteria.

| **DRUG_NAME** | **THERAPEUTIC_CATEGORY** |
| --- | --- |
| chlorpromazine | Antipsychotics-First generation |
| fluphenazine | Antipsychotics-First generation |
| haloperidol | Antipsychotics-First generation |
| loxapine | Antipsychotics-First generation |
| molindone | Antipsychotics-First generation |
| pimozide | Antipsychotics-First generation |
| prochlorperazine | Antipsychotics-First generation |
| thioridazine | Antipsychotics-First generation |
| thiothixene | Antipsychotics-First generation |
| trifluoperazine | Antipsychotics-First generation |
| perphenazine | Antipsychotics-First generation |
| asenapine | Antipsychotics-Second generation |
| clozapine | Antipsychotics-Second generation |
| iloperidone | Antipsychotics-Second generation |
| lumateperone | Antipsychotics-Second generation |
| lurasidone | Antipsychotics-Second generation |
| paliperidone | Antipsychotics-Second generation |
| quetiapine | Antipsychotics-Second generation |
| risperidone | Antipsychotics-Second generation |
| ziprasidone | Antipsychotics-Second generation |
| cariprazine | Antipsychotics-Second generation |
| aripiprazole | Antipsychotics-Second generation |
| brexpiprazole | Antipsychotics-Second generation |
| pimavanserin | Antipsychotics-Second generation |
| olanzapine | Antipsychotics-Second generation |
| alprazolam | CNS-Benzodiazepines, short acting |
| chlordiazepoxide | CNS-Benzodiazepines, short acting |
| clorazepate | CNS-Benzodiazepines, short acting |
| lorazepam | CNS-Benzodiazepines, short acting |
| oxazepam | CNS-Benzodiazepines, short acting |
| diazepam | CNS-Benzodiazepines, short acting |
| clonazepam | CNS-Benzodiazepines, long acting |
| estazolam | CNS-Benzodiazepines, long acting |
| quazepam | CNS-Benzodiazepines, long acting |
| temazepam | CNS-Benzodiazepines, long acting |
| triazolam | CNS-Benzodiazepines, long acting |
| eszopiclone | CNS-Non-Benzodiazepines |
| zaleplon | CNS-Non-Benzodiazepines |
| zolpidem | CNS-Non-Benzodiazepines |
| meperidine | Pain |

**Supplementary Appendix 3:** Demographics of Training and Test Sample

|  | **Training Sample**  **(N=264,086)** | **Test Sample**  **(N=131,906)** |
| --- | --- | --- |
|  | **Mean (SD)** | |
| **Age** | 80.59 (7.36) | 80.586 (7.35) |
| **COPS 2.5 (comorbidity score)** | 45.142 (38.57) | 45.124 (38.55) |
| **# of ED + hospital days in previous 90 days** | 2.167 (4.79) | 2.195 (4.87) |
| **# of ED Treat and Release in previous 90 days** | 0.548 (1.55) | 0.554 (1.56) |
| **Number of medication fills in previous 90 days** | 8.538 (6.92) | 8.548 (6.92) |

|  | **Training Sample**  **(N=264,086)** | | **Test Sample**  **(N=131,906)** |
| --- | --- | --- | --- |
|  | **N (% of Total)** | | |
| **abLAPS (lab acuity score)** | | | |
| HIGH | 50,105 (19.0) | 25,290 (19.2) | |
| LOW | 20,923 (7.9) | 10,400 (7.9) | |
| MED | 20,350 (7.7) | 9,959 (7.6) | |
| MISSING | 172,708 (65.4) | 86,257 (65.4) | |
| **History of Dementia** | | | |
| No | 207,664 (78.6) | 103,508 (78.5) | |
| Yes | 56,422 (21.4) | 28,398 (21.5) | |
| **History of Delerium** | | | |
| No | 209,645 (79.4) | 104,669 (79.4) | |
| Yes | 54,441 (20.6) | 27,237 (20.6) | |
| **High Risk Medications*** | | | |
| No | 223,674 (84.7) | 111,554 (84.6) | |
| Yes | 40,412 (15.3) | 20,352 (15.4) | |
| **kp.org Patient Portal Use** | | | |
| Active | 188,375 (71.3) | 94,064 (71.3) | |
| Not Active | 75,711 (28.7) | 37,842 (28.7) | |
| **Neurologic assessment in previous 90 days Flag** | | | |
| No | 251,818 (95.4) | 125,850 (95.4) | |
| Yes | 12,268 (4.6) | 6,056 (4.6) | |
| **PCP Assigned** | | | |
| No | 4,534 (1.7) | 2,286 (1.7) | |
| Yes | 259,552 (98.3) | 129,620 (98.3) | |
| **Prior level of function (PLOF)** | | | |
| Able to Walk | 83,449 (31.6) | 41,741 (31.6) | |
| No Score | 167,320 (63.4) | 83,508 (63.3) | |
| Unable to Walk | 13,317 (5.0) | 6,657 (5.0) | |
| **Schmid Falls Risk** | | | |
| High Fall Risk | 46,244 (17.5) | 23,217 (17.6) | |
| Low Fall Risk | 112,551 (42.6) | 56,110 (42.5) | |
| No Score | 105,291 (39.9) | 52,579 (39.9) | |
| **Prior substance abuse** | | | |
| No | 252,017 (95.4) | 125,868 (95.4) | |
| Yes | 12,069 (4.6) | 6,038 (4.6) | |
| **Social Determinants of Health: Any Flag **** | | | |
| No | 261,558 (99.0) | 130,660 (99.1) | |
| Yes | 2,528 (1.0) | 1,246 (0.9) | |
| **Social Determinants of Health: Family Flag **** | | | |
| No | 263,330 (99.7) | 131,512 (99.7) | |
| Yes | 756 (0.3) | 394 (0.3) | |
| **Social Determinants of Health: Finances Flag **** | | | |
| No | 263,226 (99.7) | 131,487 (99.7) | |
| Yes | 860 (0.3) | 419 (0.3) | |
| **Social Determinants of Health: Social Support Flag **** | | | |
| No | 263,121 (99.6) | 131,440 (99.6) | |
| Yes | 965 (0.4) | 466 (0.4) | |
| **Outcome: 3 or more contact days or death in next 90 days** | | | |
| No | 179,017 (67.8) | 89,571 (67.9) | |
| Yes | 85,069 (32.2) | 42,335 (32.1) | |

* High Risk medications were defined as based on a subset of Beers Criteria medications including antipsychotics and benzodiazepines (Supplement B) prescribed or active in the 90 days preceding the ED visit.

** Social Determinants of Health Flags were based on ICD-10 codes grouped into categories of family, finances, and social support (Supplement A)

**Supplementary Appendix 4:** Calibration plots for each model based on Test dataset

**
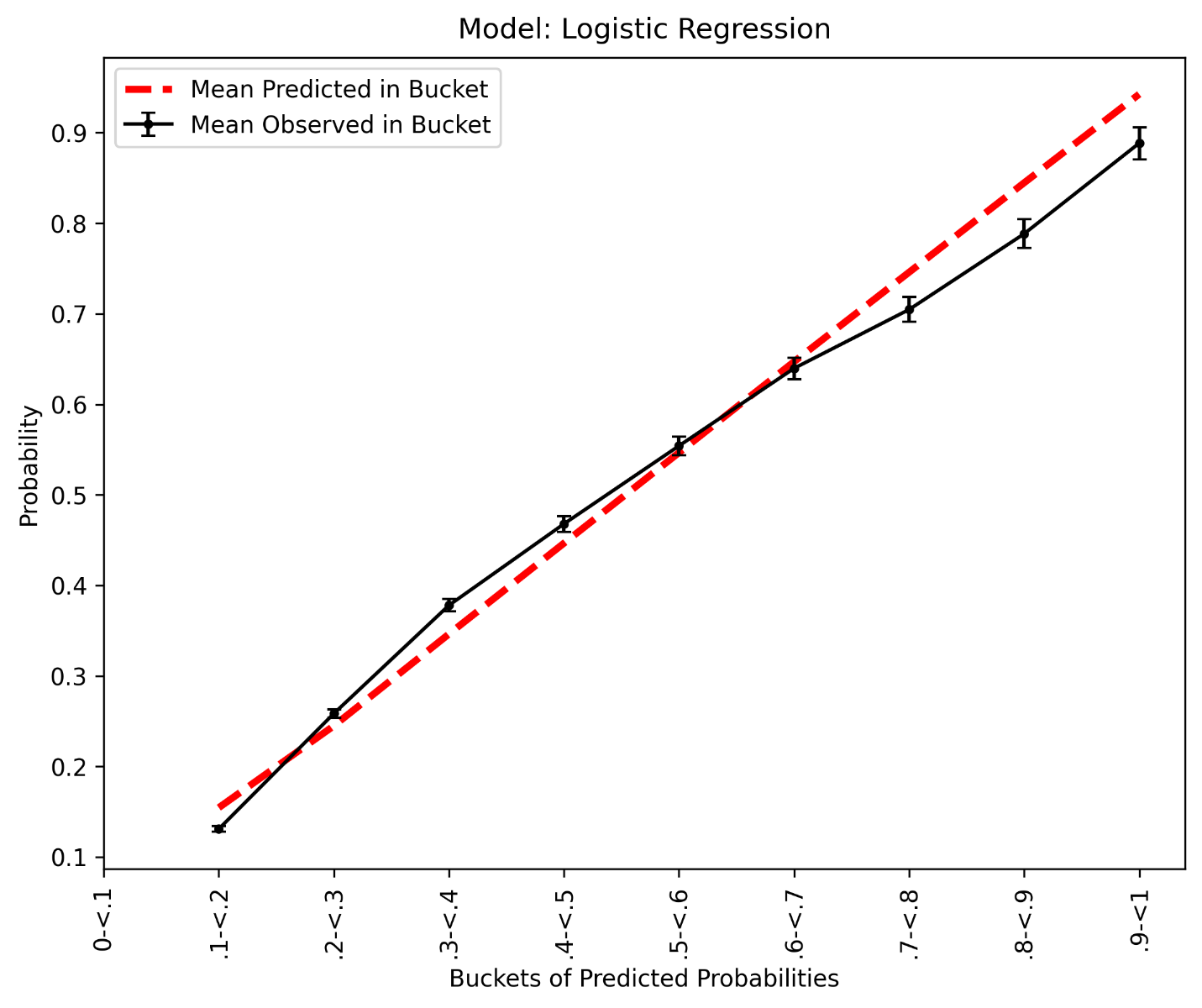
**

**
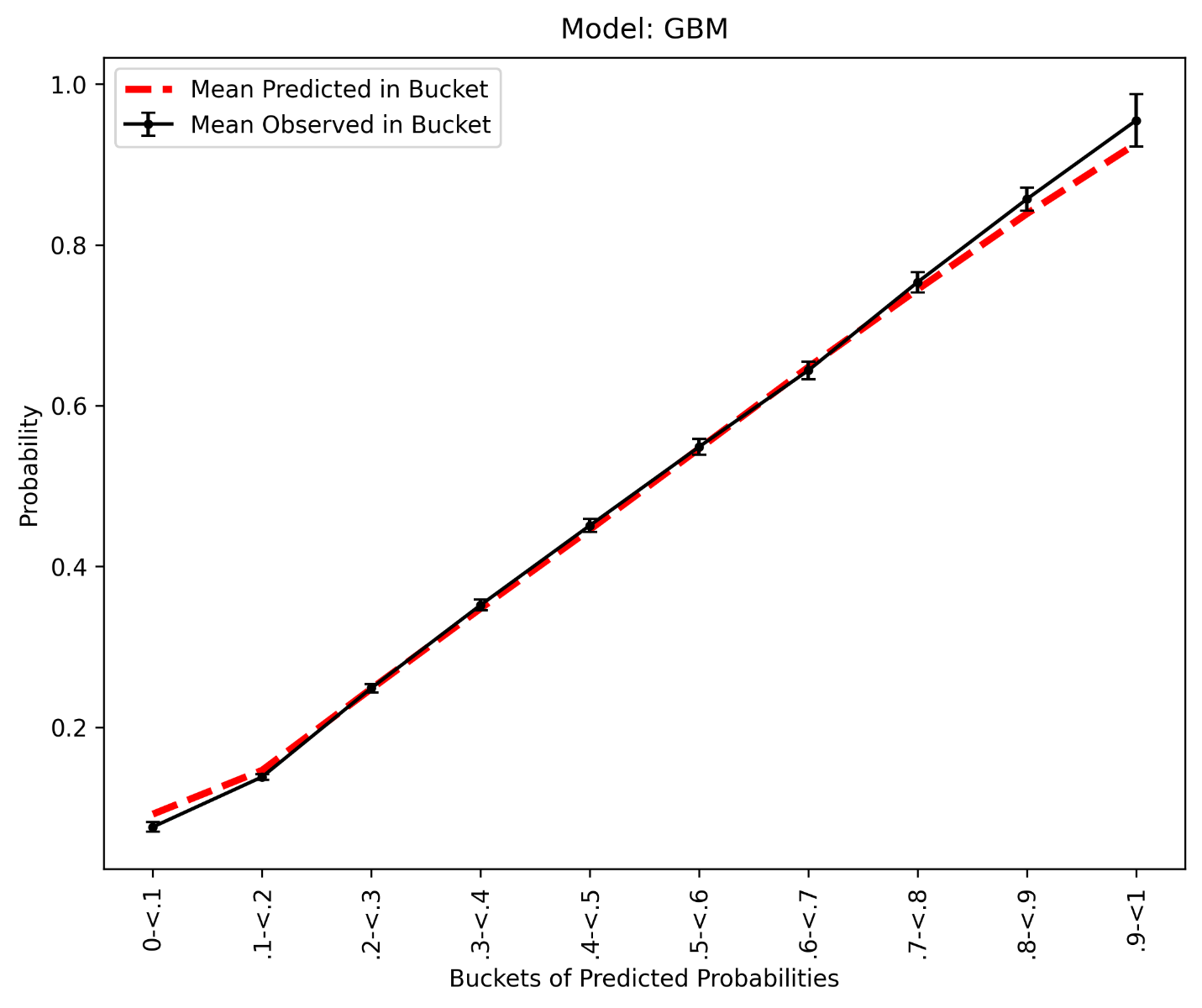
**

**
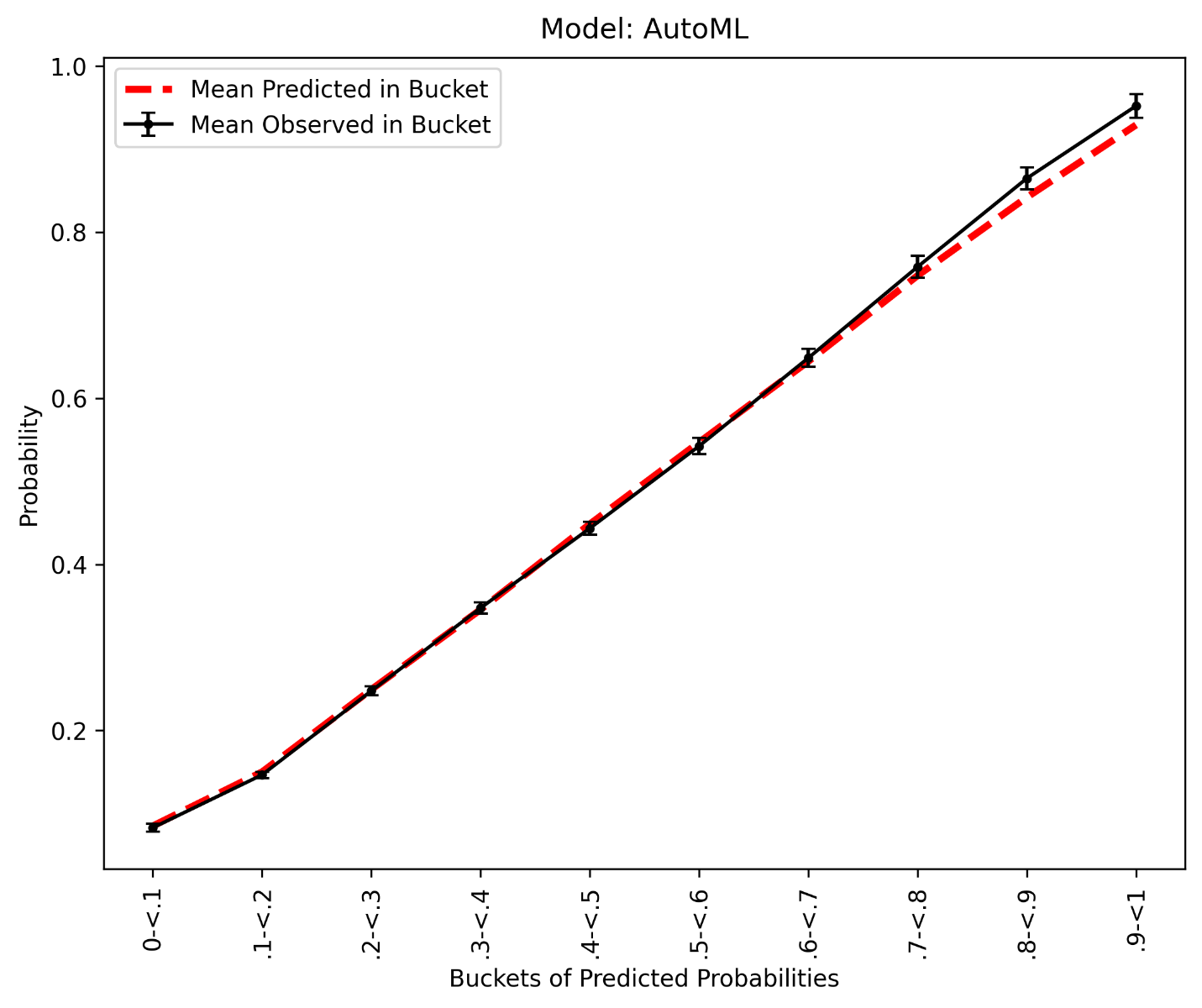
**

**
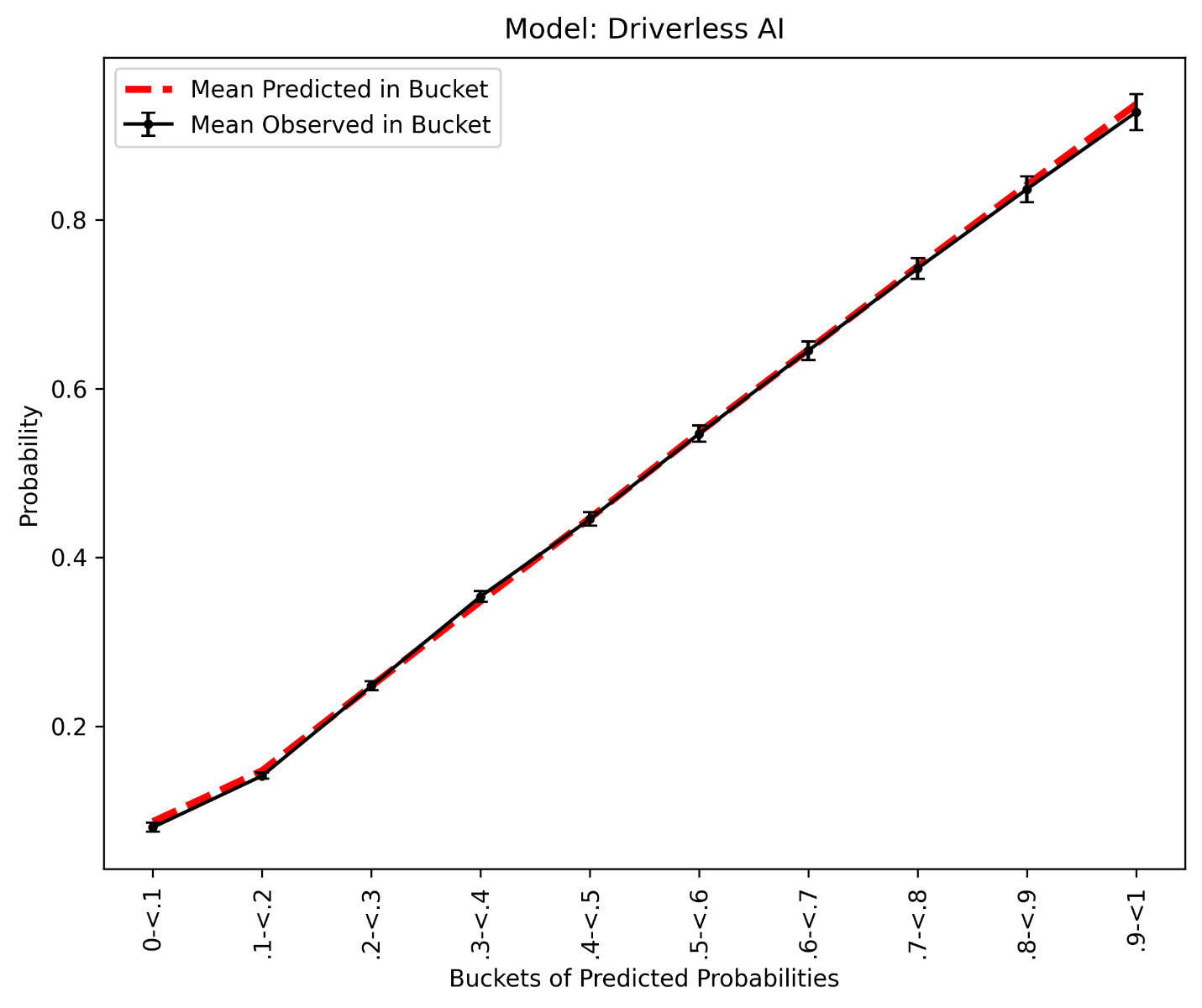
**

**Supplementary Appendix 5:** Results by Race/Ethnicity, Age Group, and Sex

AUC by Category

|  |  | |  | | **AUC** | | |
| --- | --- | --- | --- | --- | --- | --- | --- |
|  | **Population Size** | | **Target Prevalence** | | **GBM** | **GLM** | **AutoML** |
| **Race/Ethnicity** | |  | |  |  |  |  |
| All | 131906 (100%) | | 42335 (32.1%) | | 0.751 | 0.748 | 0.755 |
| Asian | | 13077 (9.9%) | | 3923 (30%) | 0.771 | 0.767 | 0.774 |
| White | | 80263 (60.8%) | | 26006 (32.4%) | 0.742 | 0.739 | 0.746 |
| Hispanic | | 18379 (13.9%) | | 5639 (30.7%) | 0.758 | 0.752 | 0.76 |
| Black/African American | | 10921 (8.3%) | | 3879 (35.5%) | 0.769 | 0.765 | 0.773 |
| Other | 9266 (7%) | | 2888 (31.2%) | | 0.767 | 0.763 | 0.771 |
| **Age Group** |  | |  | |  |  |  |
| 70-75 | 34072 (25.8%) | | 9161(26.9%) | | 0.793 | 0.79 | 0.796 |
| 75-80 | 30204 (22.9%) | | 8849(29.3%) | | 0.761 | 0.758 | 0.764 |
| 80-85 | 27129 (20.6%) | | 9010(33.2%) | | 0.736 | 0.733 | 0.739 |
| 85+ | 40501 (30.7%) | | 15315(37.8%) | | 0.692 | 0.688 | 0.697 |
| All | 131906 (100%) | | 42335(32.1%) | | 0.751 | 0.748 | 0.755 |
| **Sex** |  | |  | |  |  |  |
| Male | 56701 (43%) | | 19778 (34.9%) | | 0.745 | 0.741 | 0.75 |
| Female | 75204 (57%) | | 22557 (30%) | | 0.755 | 0.752 | 0.758 |
| All | 131905 (100%) | | 42335 (32.1%) | | 0.751 | 0.748 | 0.755 |

GLM Performance @ F1-Max

|  | **N** | **Flagged** | **True Positives** | **NNE** | **Sensitivity (%)** | **Specificity (%)** |
| --- | --- | --- | --- | --- | --- | --- |
| **Race/Ethnicity** |  |  |  |  |  |  |
| All | 131906 | 63961 (48.5%) | 30967 | 2.1 | 73.1 | 63.2 |
| Asian | 13077 | 5366 (41%) | 2700 | 2 | 68.8 | 70.9 |
| White | 80263 | 39277 (48.9%) | 18849 | 2.1 | 72.5 | 62.4 |
| Hispanic | 18379 | 8821 (48%) | 4160 | 2.1 | 73.8 | 63.4 |
| Black/African American | 10921 | 5888 (53.9%) | 3063 | 1.9 | 79.0 | 59.9 |
| Other | 9266 | 4609 (49.7%) | 2195 | 2.1 | 76.0 | 62.2 |
| **Age Group** |  |  |  |  |  |  |
| 70-75 | 34072 | 11030 (32.4%) | 5979 | 1.8 | 65.3 | 79.7 |
| 75-80 | 30204 | 12442 (41.2%) | 6123 | 2 | 69.2 | 70.4 |
| 80-85 | 27129 | 13886 (51.2%) | 6626 | 2.1 | 73.5 | 59.9 |
| 85+ | 40501 | 26603 (65.7%) | 12239 | 2.2 | 79.9 | 43 |
| All | 131906 | 63961 (48.5%) | 30967 | 2.1 | 73.1 | 63.2 |
| **Sex** |  |  |  |  |  |  |
| Male | 56701 | 28880 (50.9%) | 14619 | 2 | 73.9 | 61.4 |
| Female | 75204 | 35081 (46.6%) | 16348 | 2.1 | 72.5 | 64.4 |
| All | 131905 | 63961 (48.5%) | 30967 | 2.1 | 73.1 | 63.2 |

For all models across all subgroups, the AUC ranged from a minimum of .739 to a maximum of .774. At a specific optimal threshold for the logistic regression model, the sensitivity was highest for the Black/African American group (79%) and lowest for Asian (69%). The results were broadly similar across Age Group and Sex categories as well. For the Age Groups, the 85+ category had the lowest AUC (0.69) and lower specificity (43.0%) compared to the other groups.

**Supplementary Appendix 6:** Deciles of Thresholds for Logistic Regression Model (Test dataset)

| **Thresholds** | **% Flag** | **Sensitivity** | **Specificity** | **NNE** | **PPV** | **F1** |
| --- | --- | --- | --- | --- | --- | --- |
| 0.1 | 100.0% | 1 | 0 | 3.12 | 0.32 | 0.49 |
| 0.2 | 65.4% | 0.86 | 0.44 | 2.37 | 0.42 | 0.57 |
| 0.3 | 41.8% | 0.67 | 0.7 | 1.95 | 0.51 | 0.58 |
| 0.4 | 27.4% | 0.5 | 0.83 | 1.71 | 0.58 | 0.54 |
| 0.5 | 17.8% | 0.36 | 0.91 | 1.54 | 0.65 | 0.46 |
| 0.6 | 10.9% | 0.24 | 0.95 | 1.42 | 0.71 | 0.36 |
| 0.7 | 6.1% | 0.14 | 0.98 | 1.32 | 0.76 | 0.24 |
| 0.8 | 2.9% | 0.07 | 0.99 | 1.22 | 0.82 | 0.13 |
| 0.9 | 0.9% | 0.02 | 1 | 1.13 | 0.89 | 0.05 |
| 1 | 0.0% | 0 | 1 | 1 | 1 | 0 |
